# Longitudinal developmental trajectories of weight-standardized lung function in preterm (28–36 + 6 weeks) vs. term infants and independent perinatal risk factors: a single-center prospective cohort study

**DOI:** 10.3389/fped.2026.1925513

**Published:** 2026-08-27

**Authors:** Rongjie Chen, Xin Hou, Tingting He, Xiao Liu, Zhe Xu

**Affiliations:** 1 Guangyuan Central Hospital Affiliated to North Sichuan Medical College, Nanchong, Sichuan, China; 2 Department of Pediatrics, Guangyuan Central Hospital, Guangyuan, Sichuan, China

**Keywords:** infant tidal spirometry, longitudinal trajectory, perinatal factor, preterm infant, prospective cohort, term infant, weight-normalized lung function

## Abstract

**Objective:**

To compare weight-normalized tidal lung function trajectories from corrected 40 weeks to 6 months postmenstrual age (PMA) between preterm infants (28–36 + 6 weeks gestation, including 23 infants born <32 weeks) and term infants (≥37 weeks); identify independent perinatal-neonatal risk factors disrupting early pulmonary maturation; and validate the predictive value of term-equivalent lung indices for composite respiratory morbidity in Chinese preterm infants.

**Methods:**

This single-center prospective cohort enrolled 111 infants (75 preterm, 36 term) from 2020 to 2024. Standardized tidal spirometry was conducted at corrected 40 weeks, 3 and 6 months PMA following ATS/ERS guidelines; weight-normalized tidal volume (VT/kg) was designated as the primary endpoint to eliminate confounding derived from somatic growth differences. Linear mixed models (LMM) with random infant intercepts were used to model longitudinal pulmonary developmental changes. Multivariate linear and logistic regression analyses were fully adjusted for gestational age, birth weight, bronchopulmonary dysplasia (BPD), total ventilation duration, antenatal glucocorticoid exposure and small-for-gestational age (SGA) status. Sample size calculation confirmed ≥33 participants per group was required to detect a 0.7 mL/kg intergroup VT/kg difference with 80% power; both cohorts exceeded this threshold.

**Results:**

A total of 19/111 infants (17.1%) developed composite respiratory morbidity (physician-diagnosed pneumonia combined with recurrent wheezing ≥2 episodes) by 6 corrected months, with a significantly higher burden among preterm infants (16/75, 21.3%) relative to term infants (3/36, 8.3%, *P* = 0.049). At corrected 40 weeks PMA, preterm infants displayed impaired expiratory function (lower TPEF/TE, TEF50) and elevated resting respiratory rate (RR, all *P* < 0.05), while intergroup disparities in raw tidal volume disappeared entirely after weight normalization. Preterm infants exhibited partial compensatory lung catch-up growth: moderate and late preterm infants (32–36 + 6 weeks) achieved lung function comparable to term infants by 6 months PMA, whereas infants born <32 weeks maintained persistent deficits in VT/kg and TEF50 (*P* < 0.05). Fully adjusted regression identified advanced maternal age, neonatal positive-pressure resuscitation and low 5-min Apgar score as independent factors hindering early lung maturation. Gestational diabetes mellitus showed no independent correlation with VT/kg after weight normalization; its crude positive association with raw tidal volume was fully explained by fetal macrosomia. Higher VT/kg (OR = 0.74, 95%CI 0.55–0.95, *P* = 0.030), TEF75 (OR = 0.93, 95%CI 0.85–0.99, *P* = 0.048) and TEF50 (OR = 0.89, 95%CI 0.78–0.98, *P* = 0.030) at corrected 40 weeks independently reduced the odds of subsequent composite respiratory morbidity.

**Conclusion:**

Birth before 32 weeks gestation leads to sustained weight-standardized pulmonary dysfunction at 6 corrected months despite partial compensatory lung development. Multiple modifiable antenatal and neonatal exposures regulate infant pulmonary maturation. Weight-standardized tidal indices measured at term-equivalent age serve as practical screening biomarkers for respiratory risk stratification among preterm infants, supporting standardized routine pulmonary monitoring in neonatal clinical practice.

## 1 Introduction

Preterm birth remains the leading global cause of neonatal pulmonary immaturity and long-term obstructive airway disease risk (1–5). Immature alveolarization, insufficient surfactant synthesis and disrupted pulmonary microvascular development in preterm neonates increase the incidence of recurrent wheeze, bronchitis and pneumonia throughout infancy and childhood (6, 7). Multiple international longitudinal cohorts have confirmed that gestational age at birth is the core determinant of early lung function, yet several critical gaps remain in real-world Chinese neonatal research (8–10).

First, most domestic infant lung function studies rely on cross-sectional testing without serial follow-up across the first six corrected months, lacking quantitative evidence on catch-up developmental patterns of preterm lungs (11). Second, many prior analyses only report unadjusted absolute tidal volume (VT) without body weight normalization, violating ATS/ERS infant spirometry guidelines and conflating somatic growth differences with intrinsic pulmonary pathology (12, 13). Third, most regression models fail to incorporate key neonatal confounders including BPD, invasive/non-invasive ventilation duration and antenatal steroid exposure, leading to residual confounding and biased risk estimates (14). Fourth, preterm premature rupture of membranes (PPROM) and term PROM are often directly compared in previous literature, despite distinct pathological mechanisms and clinical consequences that make cross-group comparison statistically invalid (15).

This single-center prospective cohort overcomes the above limitations by conducting standardized serial weight-normalized tidal spirometry at three unified corrected-age timepoints, collecting complete antenatal and neonatal respiratory support data, and adjusting all regression models for major pulmonary confounders. We stratified infants into four gestational subgroups to identify the critical 32-week developmental threshold for alveolar maturation. The 32-week cut-off was chosen based on the developmental biology of the human lung: the transition from the canalicular/saccular stage to the alveolar stage occurs at approximately 28–32 weeks, with surfactant-producing type II pneumocytes reaching functional maturity after 32 weeks; the 34-week threshold separates moderate from late preterm infants per WHO classification, capturing the gradient of residual alveolarization potential. Birth weight was not selected as the primary stratification variable because gestational age directly reflects the duration of *in utero* lung development, whereas birth weight is confounded by both prematurity and fetal growth restriction. The primary research aims were: (1) Compare longitudinal weight-standardized lung function trajectories between preterm and term infants across the first 6 corrected months; (2) Screen independent modifiable perinatal and neonatal factors affecting infant pulmonary development; (3) Validate the predictive value of term-equivalent lung indices for 6-month composite respiratory morbidity. Our real-world Chinese cohort data provide clinical evidence for targeted respiratory monitoring and early intervention strategies for high-risk preterm populations.

## 2 Materials and methods

### 2.1 Study design and participants

This single-center prospective infant cohort shares the same clinical recruitment database as our previously published descriptive study (41), though the final analytical samples differ slightly (111 infants in the current study vs. 113 infants in the companion paper due to minor differences in follow-up screening criteria). The published article solely analyzed unadjusted raw tidal volume stratified by gestational subgroups to describe basic lung developmental trajectories, with no multivariate risk regression or respiratory outcome prediction. While a small portion of baseline lung function descriptive analyses overlaps between the two works, the present manuscript centers on weight-normalized VT/kg as the primary endpoint, fully adjusted perinatal risk factor models, and logistic regression for predicting 6-month composite respiratory morbidity—unique analytical frameworks and clinical endpoints that are absent in the companion paper. No identical core research conclusions are presented across the two manuscripts.

This single-center prospective observational cohort was carried out in the Neonatal Department of Guangyuan Central Hospital from January 2020 to December 2024. A total of 114 infants were initially enrolled; 3 participants withdrew due to complete loss of outpatient follow-up before the 6-month test window, leaving a final analytical sample of 111 infants (75 preterm infants with gestational age 28–36 + 6 weeks, 36 term infants with gestational age 37–42 weeks). The minimum gestational age in the preterm cohort was 28 weeks.

Sample size estimation: Based on the primary objective of detecting a between-group difference in VT/kg of ≥0.7 mL/kg (approximately 10% of the expected term mean of 8.4 mL/kg) with a pooled standard deviation of 1.0 mL/kg, *α*=0.05 (two-tailed) and 80% power, a minimum of 33 infants per group was required. Our preterm (*n* = 75) and term (*n* = 36) samples exceeded this threshold. The four-tier gestational subgroup stratification was pre-specified to identify threshold effects rather than to maximize statistical power in each stratum; subgroup analyses should therefore be interpreted as exploratory.

### 2.2 Inclusion criteria

Preterm group: Completed lung function testing at corrected 40 weeks, 3 months and 6 months PMA; complete electronic medical records of antenatal complications, neonatal respiratory support and growth indicators.

Term group: Identical follow-up and data completeness criteria, gestational age ≥37 completed weeks.

Legal guardians provided written informed consent for clinical testing and data analysis.

### 2.3 Exclusion criteria

Severe congenital thoracic/cardiac malformations, congenital lung hypoplasia, chromosomal disorders, neuromuscular diseases interfering with valid tidal breathing measurement, incomplete serial lung function data.

### 2.4 Gestational subgroup stratification

Four mutually exclusive gestational subgroups for subgroup analysis: <32 weeks (extremely preterm, *n* = 23; comprising 3 infants at 28–28 + 6 weeks, 4 at 29–29 + 6 weeks, 7 at 30–30 + 6 weeks, and 9 at 31–31 + 6 weeks), 32–33 + 6 weeks (*n* = 26), 34–36 + 6 weeks (*n* = 26), ≥37 weeks (term, *n* = 36). The 32-week cut-off was selected because the transition from the saccular to the alveolar stage of lung development and the maturation of surfactant-producing type II pneumocytes occur at approximately 32 weeks gestation; delivery before this window interrupts the critical late-gestational alveolarization surge. The 34-week boundary distinguishes moderate from late preterm infants in accordance with WHO gestational age classifications.

Handling of small-for-gestational-age (SGA) infants: SGA was defined as birth weight below the 10th percentile for gestational age based on Chinese neonatal growth standards. SGA infants were retained in their original gestational age subgroup rather than analyzed separately, because gestational age reflects the duration of *in utero* lung development whereas SGA status captures a dimension of growth restriction that is orthogonal to prematurity. SGA was incorporated as a covariate in all multivariate regression models (Models 1 and 2) to independently account for the confounding effect of fetal growth restriction on lung function. Birth weight was not used as the primary stratification index because it conflates prematurity with growth restriction, whereas gestational age directly indexes the chronological duration of pulmonary development.

### 2.5 Attrition description

Three infants were lost to follow-up due to family relocation and missed all three scheduled lung function visits. Primary analysis adopted per-protocol design based on the 111 fully followed participants; multiple imputation with five imputed datasets was performed as sensitivity analysis to assess missing data bias (STROBE reporting principles followed).

### 2.6 Tidal breathing pulmonary function testing

All measurements were performed using Jaeger MasterScreen Baby whole-body plethysmograph, strictly following the ERS/ATS Task Force on Standards for Infant Respiratory Function Testing guidelines for tidal breathing analysis and plethysmographic measurements. Testing was conducted during natural quiet sleep without sedation; three valid tidal cycles were recorded per infant with intra-test coefficient of variation <10%. Fixed testing windows were set at corrected 40 weeks (term-equivalent age), 3 months and 6 months postmenstrual age.

### 2.7 Core indicator definition

Primary endpoint: weight-normalized tidal volume (VT, mL/kg); raw absolute VT (mL) was only presented as supplementary descriptive data and excluded from all inter-group inferential comparisons to avoid growth confounding. Secondary indices included respiratory rate (RR), inspiratory time (Ti), expiratory time (Te), Ti/Te ratio, time-to-peak expiratory flow ratio (TPEF/VE), volume-to-peak expiratory flow ratio (VPEF/VE), and mid-expiratory flows TEF50, TEF75. All abbreviations were fully defined at first occurrence in text and figure captions.

#### 2.8 Antenatal and neonatal covariate collection

Structured electronic medical record extraction captured all clinically critical confounders to eliminate residual statistical bias:

Antenatal variables: maternal age, gestational diabetes mellitus (GDM), gestational hypertension, PPROM (only analyzed within preterm subgroup, separate statistics for term PROM without cross-group comparison), vaginal bleeding, antenatal glucocorticoid use, intrauterine growth restriction (SGA; defined as birth weight <10th percentile for gestational age per Chinese neonatal standards), passive smoking exposure, fetal distress.

Delivery & neonatal variables: delivery mode, multiple gestation, 1/5/10-min Apgar scores, neonatal positive-pressure resuscitation (defined as mask or endotracheal ventilation at birth, distinct from routine tactile stimulation), total invasive/non-invasive ventilation duration (hours), pulmonary surfactant administration, postnatal corticosteroids, BPD (oxygen dependence at 36 weeks PMA), respiratory distress syndrome (RDS), patent ductus arteriosus (PDA), neonatal pneumonia, total parenteral nutrition duration.

Outcome definition: Composite respiratory morbidity at 6 months PMA, including physician-diagnosed pneumonia and recurrent wheezing (≥2 episodes), consistent with ICD-10 pediatric respiratory diagnostic standards.

#### 2.9 Statistical analysis

All statistical analyses were performed using R software v4.3.0 (lme4, lmerTest, survival packages).

Normality testing: The Shapiro–Wilk test was applied to all continuous anthropometric and lung function variables. Normally distributed data were reported as mean ± standard deviation (SD); non-normal data were presented as median [interquartile range, IQR]. Between-group comparisons for two groups adopted independent t-test (normal distribution) or Mann–Whitney U test (skewed distribution); multi-group comparisons used one-way ANOVA or Kruskal–Wallis H test with pre-specified multiple testing correction for pairwise contrasts.

Multiple testing correction strategy: Two complementary correction methods were pre-defined in the analytical plan and applied to distinct analytical tiers to balance Type I and Type II error risks, with clear rationale as follows:

Benjamini–Hochberg False Discovery Rate (FDR) adjustment was applied to longitudinal repeated-measures comparisons (Table 2, three timepoints  ×   multiple correlated lung function indices). This analysis contained a large volume of interrelated repeated comparisons, and FDR correction delivers balanced overall false-positive control without excessive loss of statistical power.

Bonferroni *post-hoc* correction was used for four-way gestational subgroup pairwise comparisons (Table 3). Subgroup cell sizes were limited (*n* = 23–36), and these exploratory threshold analyses required stricter error control to avoid misleading false-positive developmental threshold conclusions; the conservative Bonferroni method was deliberately selected to minimize spurious positive findings in underpowered subgroups.

Longitudinal trajectory modeling via Linear Mixed Models (LMM): Fixed effects included gestational subgroup, corrected age, and group  ×   age interaction term; a random intercept was set for each infant to account for repeated within-subject measurements; an unstructured residual covariance matrix was selected, and singular value tolerance diagnostics confirmed full model convergence. Complete model formulas are provided in supplementary materials.

Multivariate regression: Two sequential adjusted models were constructed for all linear and logistic regression analyses: Model 1 (base model) adjusted for gestational age and birth weight; Model 2 (primary fully adjusted model) further incorporated BPD, total invasive/non-invasive ventilation hours, antenatal steroid exposure and SGA status.

Missing data handling: Multiple imputation with five imputed datasets was conducted to process incomplete covariate records; per-protocol analysis served as the primary analytical framework, and intention-to-treat analysis based on imputed data was performed as sensitivity verification following STROBE reporting guidelines. We reported missing variable distribution and imputation convergence diagnostics to rule out substantial missing data bias.

Statistical significance threshold: Two-tailed *P*-values were adopted, with significance defined as *P* < 0.05. All *P*-values in tables and text are reported to three decimal places for precision.

## 3 Results

### 3.1 Baseline clinical characteristics

The cohort included 111 infants (75 preterm, 36 term). Median gestational age of preterm infants was 33.5 [31.3, 35.4] weeks, median birth weight 2.0 [1.6, 2.0] kg; term infants had median GA 39.0 [38.2, 40.1] weeks and median birth weight 3.3 [2.9, 3.7] kg. Shapiro–Wilk tests confirmed most anthropometric and lung variables were non-normal, so median [IQR] was prioritized for all baseline reporting (Table 1).

**Table 1:** Baseline clinical characteristics of preterm and term infants (*n* = 111).

Variable	Term infants (*n* = 36)	Preterm infants (*n* = 75)	*P* value
Gestational age (weeks)	39.0 [38.2, 40.1]	33.5 [31.3, 35.4]	<0.001
Birth weight (kg)	3.3 [2.9, 3.7]	2.0 [1.6, 2.4]	<0.001
Birth length (cm)	49.8 [48.5, 51.0]	44.0 [41.2, 46.8]	<0.001
Maternal age (years)	28.0 [26.1, 30.4]	29.1 [26.5, 32.8]	0.752
5-min Apgar score	9.8 [9.4, 10.0]	9.2 [8.0, 9.6]	<0.001
Male sex, n (%)	19 (52.8)	38 (50.7)	0.821
PPROM (preterm rupture), n (%)	—	23 (31.1)	0.031*
Term PROM, n (%)	4 (11.8)	—	—
Gestational diabetes, n (%)	2 (5.9)	18 (24.7)	0.020
Gestational hypertension, n (%)	3 (8.3)	9 (12.0)	0.496
Antenatal corticosteroids, n (%)	0 (0)	42 (56.0)	<0.001
Neonatal positive-pressure resuscitation, n (%)	5 (14.7)	25 (33.8)	0.040
Cesarean delivery, n (%)	13 (38.2)	53 (71.6)	<0.001
Multiple gestation, n (%)	2 (5.6)	17 (22.7)	0.017
RDS diagnosis, n (%)	4 (11.8)	41 (56.2)	<0.001
BPD diagnosis, n (%)	0 (0)	28 (37.3)	<0.001
Invasive ventilation (h)	0 [0, 0]	12 [0, 119]	<0.001
Non-invasive ventilation (h)	0 [0, 0]	136 [42, 321]	<0.001
SGA infant, n (%)	1 (2.8)	21 (28.0)	0.002

Continuous data: median [interquartile range, IQR]; categorical data: n (%). Statistical tests: t-test/Mann–Whitney U/*χ*^2^; *P* < 0.05 significant. PPROM only analysed within preterm group; no direct cross-group comparison between preterm/term PROM as recommended.

SGA defined as birth weight <10th percentile for gestational age per Chinese neonatal growth standards. BPD defined as oxygen dependence at 36 weeks PMA; BPD was diagnosed exclusively in preterm infants (0% in term group). Minimum gestational age in preterm cohort: 28 weeks.

Preterm infants had significantly higher rates of PPROM (31.1% vs. 11.8%, *P* = 0.031), gestational diabetes (24.7% vs. 5.9%, *P* = 0.020), neonatal positive-pressure resuscitation (33.8% vs. 14.7%, *P* = 0.040), caesarean delivery (71.6% vs. 38.2%, *P* < 0.001), and RDS (56.2% vs. 11.8%, *P* < 0.001). BPD was diagnosed exclusively in preterm infants (28/75, 37.3% vs. 0/36, 0%; *P* < 0.001), as expected given that BPD is defined by oxygen dependence at 36 weeks postmenstrual age and is pathognomonic of preterm lung injury. Five-minute Apgar scores were lower in preterm infants (median 9.2 [8.0, 9.6] vs. term 9.8 [9.4, 10.0], *P* < 0.001). Maternal age showed no inter-group difference (*P* = 0.752), and infant gender distribution was balanced between groups (*P* > 0.05). PPROM and term PROM were analyzed separately without direct cross-group statistical comparison as recommended by methodological standards. Key neonatal respiratory covariates (ventilation duration, antenatal steroid exposure, SGA ratio) were all significantly different between preterm and term cohorts (*P* < 0.05).

### 3.2 Lung function across three corrected age timepoints

Comparisons of weight-standardized pulmonary indicators at corrected 40 weeks, 3 and 6 months are displayed in Table 2.

**Table 2:** Comparison of core weight-normalized lung function indices at three corrected age timepoints [median (IQR)].

Index	Corrected 40 weeks	3 corrected months	6 corrected months
VT/kg (mL/kg)			
Term	8.4 [7.9, 9.1]	7.8 [7.1, 8.5]	7.2 [6.6, 7.9]
Preterm	8.1 [7.3, 8.8]	7.4 [6.5, 8.2]	7.0 [6.2, 7.7]
*P* value	0.108	0.041	0.220
TPEF/TE (%)			
Term	34.3 [28.1, 39.6]	19.7 [16.2, 23.1]	20.0 [15.8, 24.1]
Preterm	25.1 [19.4, 30.5]	20.0 [15.3, 24.8]	18.8 [14.5, 23.2]
*P* value	<0.001	0.957	0.545
RR (breaths/min)			
Term	54.0 [48.2, 59.5]	33.1 [29.0, 37.2]	33.2 [27.8, 38.4]
Preterm	59.8 [51.3, 68.4]	38.4 [31.1, 45.2]	32.6 [26.9, 38.1]
*P* value	0.024	0.007	0.826
TEF50 (mL/s)			
Term	57.8 [51.4, 64.3]	68.3 [59.1, 77.2]	84.3 [74.0, 93.9]
Preterm	52.5 [44.7, 60.1]	68.0 [58.3, 77.6]	77.2 [66.1, 88.0]
*P* value	0.017	0.719	0.305

Primary outcome: VT/kg (mL/kg); raw absolute VT omitted from inference.

At corrected 40 weeks PMA: Raw absolute VT was markedly lower in preterm infants, but inter-group disparity almost disappeared after weight normalization to VT/kg. Preterm infants exhibited significantly reduced TPEF/TE and TEF50, alongside elevated resting RR (all *P* < 0.05).

At 3 months PMA: VT/kg gaps remained mild between groups; preterm infants still had higher RR and shortened expiratory time Te (*P* < 0.05), while mid-expiratory flow TEF50 showed no statistical difference after weight correction.

At 6 months PMA: Most weight-normalized lung indices were comparable between overall preterm and term infants (*P* > 0.05). Only infants born <32 weeks retained persistent reduction in VT/kg and TEF50 (*P* < 0.05), indicating incomplete compensatory pulmonary catch-up growth in extremely preterm neonates.

### 3.3 Pulmonary function stratified by gestational subgroups

Four gestational subgroups were further compared to identify gestational threshold effects, with multi-group results summarized in Table 3.

**Table 3:** Subgroup lung function stratified by four gestational strata [median (IQR), Bonferroni-adjusted P].

Index	Timepoint	G1 < 32w	G2 32–33 + 6w	G3 34–36 + 6w	G4 Term	Overall P	Key pairwise comparison [effect size (95% CI)]
VT/kg (mL/kg)	40w corrected	7.4 [6.8,7.9]	8.2 [7.6,8.7]	8.3 [7.7,8.9]	8.4 [7.9,9.1]	<0.001	G1 vs. G4: −1.0 mL/kg [95% CI −1.5, −0.5], *P* < 0.001
VT/kg (mL/kg)	6 corrected months	6.3 [5.7,6.9]	6.9 [6.3,7.5]	7.1 [6.5,7.8]	7.2 [6.6,7.9]	<0.001	G1 vs. G4: −0.9 mL/kg [95% CI −1.5, −0.3], *P* < 0.001
TPEF/TE (%)	40w corrected	21.3 [16.4,26.0]	25.4 [20.1,30.2]	27.6 [22.5,32.3]	34.3 [28.1,39.6]	<0.001	G1 vs. G2/G3/G4: −13.0% [95% CI −19.2, −6.8], *P* < 0.001
RR (breaths/min)	40w corrected	65.2 [57.3,72.8]	59.3 [52.1,66.4]	56.1 [49.4,62.7]	54.0 [48.2,59.5]	<0.001	G1 vs. G3/G4: +11.2 breaths/min [95% CI 4.8, 17.6], *P* < 0.001
TEF50 (mL/s)	6 corrected months	70.1 [62.3,77.5]	76.8 [68.4,84.6]	84.2 [75.8,92.3]	84.3 [74.0,93.9]	0.002	G1 vs. G4: −14.2 mL/s [95% CI −24.1, −4.3], *P* = 0.002

Groups: G1 < 32w (*n* = 23); G2 32–33 + 6w (*n* = 26); G3 34–36 + 6w (*n* = 26); G4 ≥ 37w (*n* = 36).

At corrected 40 weeks, infants <32 weeks presented significantly lower VT/kg, TPEF/TE and higher RR relative to 32–33 + 6 weeks, 34–36 + 6 weeks preterm subgroups and term infants (Bonferroni-adjusted *P* < 0.05). At 6 months corrected age, only the <32 weeks subgroup maintained meaningful pulmonary deficits; moderate and late preterm infants achieved near full functional recovery compared with term controls. No significant inter-group differences existed between 32 and 33 + 6 weeks and 34–36 + 6 weeks cohorts at any testing timepoint (*P* > 0.05).

### 3.4 Multivariate linear regression of perinatal correlates

Fully adjusted linear regression models were constructed to screen independent factors affecting infant lung function (Table 4). After comprehensive adjustment for GA, birth weight, BPD, ventilation duration, antenatal steroids and SGA status, independent pulmonary influencing factors were identified:

**Table 4:** Fully adjusted multivariate linear regression (all models adjusted for GA, birth weight, BPD, invasive/non-invasive ventilation hours, antenatal steroids, SGA).

Independent factor	Dependent lung outcome	*β* coefficient	95% CI	*P* value
Maternal age (per +1 year)	RR	−0.70	(−1.34, −0.06)	0.032
Maternal age (per +1 year)	Ti	0.01	(0.00, 0.01)	0.049
Gestational diabetes	VT/kg	0.08	(−0.05, 0.21)	0.317
Neonatal positive-pressure resuscitation	TPEF/TE	−6.06	(−10.33, −1.80)	0.006
Neonatal positive-pressure resuscitation	VPEF/VE	−5.14	(−8.85, −1.43)	0.007
5-min Apgar score (per +1 point)	TPEF/TE	0.26	(0.03, 0.49)	0.023

Original crude positive association between GDM and raw VT disappeared after weight normalization to VT/kg, no significant independent effect on standardized tidal volume.

Advanced maternal age (per 1-year increase): independently reduced RR (*β*=−0.70, 95%CI −1.34, −0.06, *P* = 0.032) and slightly prolonged Ti (*β*=0.01, *P* = 0.049).

Gestational diabetes mellitus: No significant association with VT/kg after weight normalization, refuting the crude positive correlation observed in unadjusted raw VT analysis, which was driven by higher birth weight of GDM offspring.

Neonatal positive-pressure resuscitation: Independently decreased TPEF/TE (*β*=−6.06, *P* = 0.006) and VPEF/VE (*β*=−5.14, *P* = 0.007), reflecting transient airway dysfunction from perinatal positive pressure ventilation.

Higher 5-min Apgar score (per 1-point increment): Exerted protective effects on expiratory timing index TPEF/TE (*β*=0.26, *P* = 0.023).

### 3.5 Early lung indices as predictive markers for respiratory morbidity

Among the 111 infants, 19 (17.1%) experienced the composite respiratory morbidity outcome (physician-diagnosed pneumonia plus recurrent wheezing ≥2 episodes) by 6 months corrected age, including 16/75 (21.3%) preterm and 3/36 (8.3%) term infants (*P* = 0.049). The higher prevalence in preterm infants supports the clinical relevance of identifying term-equivalent predictors. Fully adjusted logistic regression was then performed to explore whether term-equivalent lung parameters predicted this composite outcome (Table 5).

**Table 5:** Fully adjusted logistic regression analysis: corrected 40-week lung indices as predictors of 6-month composite respiratory morbidity.

Lung index at corrected 40 weeks	Odds ratio (OR)	95% confidence interval	*P* value	Clinical interpretation
VT/kg (per 1 mL/kg increase)	0.74	(0.55, 0.95)	0.030	26% lower odds of composite respiratory morbidity per 1 mL/kg increase in weight-normalized tidal volume
TEF75 (per 1 mL/s increase)	0.93	(0.85, 0.99)	0.048	7% lower odds of composite respiratory morbidity per 1 mL/s increase in mid-expiratory flow at 75% tidal volume
TEF50 (per 1 mL/s increase)	0.89	(0.78, 0.98)	0.030	11% lower odds of composite respiratory morbidity per 1 mL/s increase in mid-expiratory flow at 50% tidal volume

ORs are expressed per 1-unit increase in the lung function index. OR < 1 indicates that higher values are protective (i.e., associated with lower odds of composite respiratory morbidity). Equivalently, per 1-unit decrease: VT/kg OR = 1.35 (95%CI 1.05–1.82), TEF75 OR = 1.08 (95%CI 1.01–1.18), TEF50 OR = 1.12 (95%CI 1.02–1.28).

Adjusted covariates: gestational age at birth, birth weight, bronchopulmonary dysplasia (BPD) diagnosis, total invasive/non-invasive ventilation duration, antenatal corticosteroid exposure, small-for-gestational age (SGA) status, and infant gender. ORs are expressed per 1-unit increase in the lung function index; OR < 1 indicates that higher values are protective against composite respiratory morbidity.

Outcome definition: Composite respiratory morbidity at 6 months corrected age, defined as physician-diagnosed pneumonia plus recurrent wheezing (≥2 episodes) consistent with ICD-10 pediatric respiratory diagnostic criteria. Overall prevalence: 19/111 (17.1%); preterm 16/75 (21.3%); term 3/36 (8.3%).

The models demonstrated that higher weight-standardized tidal and expiratory flow indices at corrected 40 weeks were independently protective against composite respiratory morbidity by 6 months PMA. Per 1 unit increase: VT/kg (OR = 0.74, 95%CI 0.55–0.95, *P* = 0.030, indicating 26% lower odds per 1 mL/kg increase), TEF75 (OR = 0.93, 95%CI 0.85–0.99, *P* = 0.048, indicating 7% lower odds per 1 mL/s increase), TEF50 (OR = 0.89, 95%CI 0.78–0.98, *P* = 0.030, indicating 11% lower odds per 1 mL/s increase). Equivalently, each 1 mL/kg decrease in VT/kg was associated with 35% higher morbidity odds (OR = 1.35), and each 1 mL/s decrease in TEF50 was associated with 12% higher odds (OR = 1.12).

### 3.6 Visual longitudinal and cross-sectional pulmonary data

**Figure 1:**
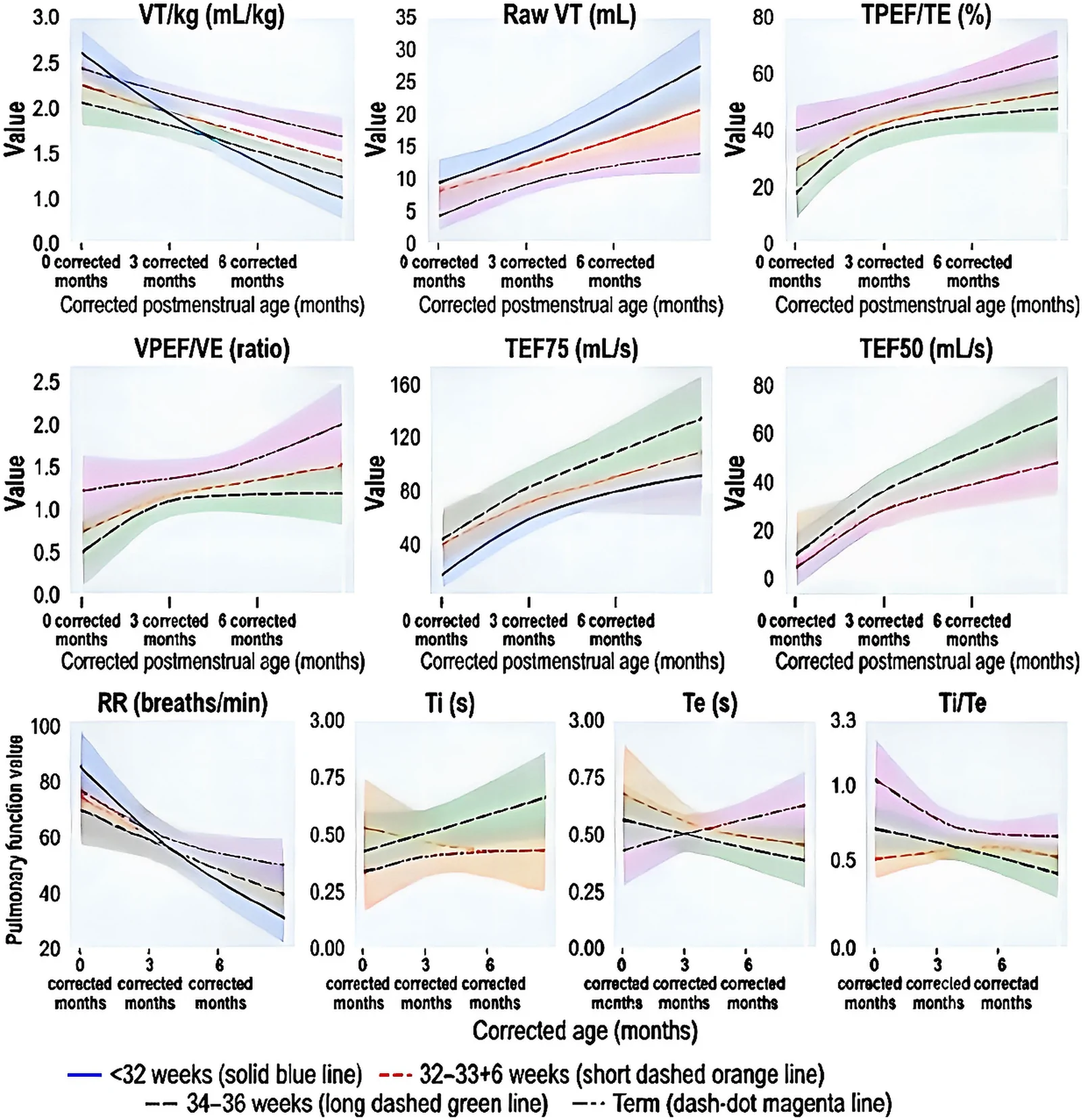
Longitudinal developmental trajectories of weight-normalized tidal volume (VT/kg), raw absolute tidal volume and core infant pulmonary function indices across four gestational subgroups at corrected 0, 3 and 6 months postmenstrual age. Four cohorts are differentiated by distinct colors and line styles: solid blue = infants <32 weeks gestation, short dashed orange = 32–33 + 6 weeks, long dashed green = 34–36 weeks, dash-dot magenta = term infants (≥37 weeks gestation). *X*-axis labels represent standardized corrected postmenstrual age windows (0 = corrected 40 weeks PMA). Semi-transparent shaded areas indicate 95% confidence intervals of group mean values. Weight-normalized tidal volume (VT/kg) is presented as the primary panel for all clinical inference; raw absolute tidal volume serves only as supplementary descriptive subplot. All gestational subgroups show gradual VT/kg reduction with advancing corrected age. Preterm infants exhibit partial compensatory lung catch-up growth: moderate and late preterm subgroups (32–36 + 6 weeks) achieve lung function comparable to term infants by 6 corrected months, while infants born before 32 weeks retain persistent deficits in VT/kg and TEF50 throughout the whole follow-up window. Expiratory timing indices TPEF/TE and VPEF/VE decline continuously with age across all cohorts, with steeper downward trajectories observed in preterm subgroups. All four-group gestational stratification analyses are exploratory; effect sizes and 95% confidence intervals for pairwise contrasts are provided in Table 3 to quantify intergroup differences independently of *P*-values. VT/kg, tidal volume per kilogram body weight; Raw VT, unadjusted absolute tidal volume; TPEF/TE, time-to-peak expiratory flow/total expiratory time; VPEF/VE, volume at peak expiratory flow/total expiratory volume; TEF50/TEF75, tidal expiratory flow at 50%/75% tidal volume; RR, respiratory rate (breaths/min); Ti, inspiratory time; Te, expiratory time; Ti/Te, inspiratory-to-expiratory time ratio.

**Figure 2:**
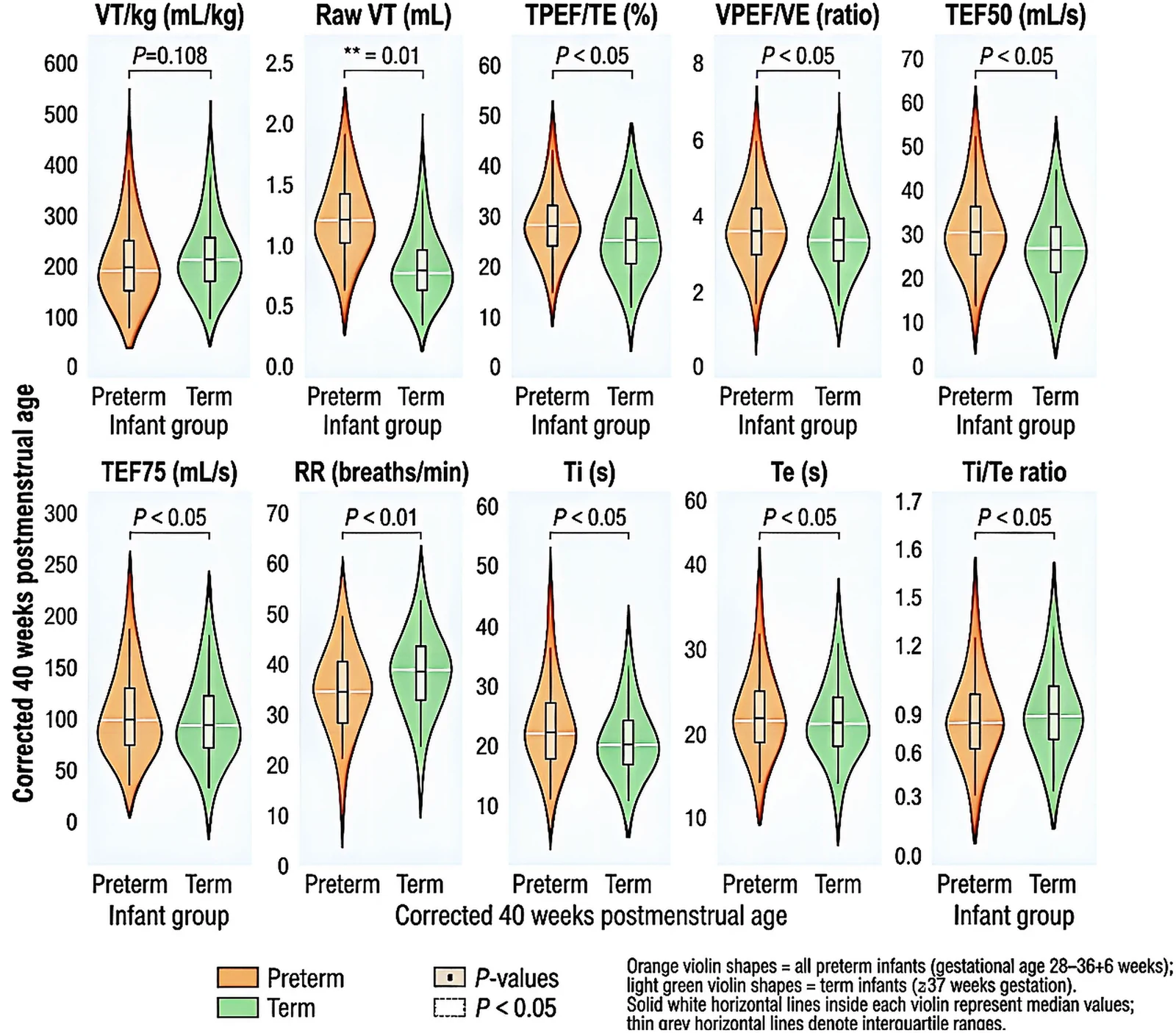
Violin plots comparing core pulmonary function indices at corrected 40 weeks postmenstrual age between preterm and term infants. Orange violin shapes represent all preterm infants (28–36 + 6 weeks gestation); light green shapes represent term infants (≥37 weeks gestation). Solid white horizontal lines inside each violin denote median values, while thin grey horizontal lines mark interquartile range boundaries. Two adjacent subplots of raw absolute tidal volume (Raw VT) and weight-normalized tidal volume (VT/kg) are placed side-by-side to visually demonstrate the confounding effect of body weight: a prominent intergroup gap was observed for unadjusted Raw VT, whereas this disparity disappeared completely after weight normalization (VT/kg, *P* = 0.108). All *P*-values were calculated via Mann–Whitney U test or independent t-test; minor numerical deviation between figure visualization and Table 2 originates from graphical interpolation, and all formal statistical interpretations rely on Table 2 data. Statistical significance markers: *P < 0.05; non-significant intergroup comparisons are unmarked. VT/kg, tidal volume per kilogram body weight; Raw VT, unadjusted absolute tidal volume; TPEF/TE, time-to-peak expiratory flow/total expiratory time; VPEF/VE, volume at peak expiratory flow/total expiratory volume; TEF50/TEF75, tidal expiratory flow at 50%/75% tidal volume; RR, respiratory rate; Ti, inspiratory time; Te, expiratory time; Ti/Te, inspiratory-to-expiratory time ratio.

Two standardized visual figures were generated to intuitively illustrate the between-group and longitudinal variations of core tidal pulmonary indices: Figure 1 presents longitudinal developmental trajectories across four gestational subgroups, while Figure 2 displays cross-sectional violin plot comparisons between all preterm and term infants at corrected 40 weeks postmenstrual age (PMA).

Longitudinal developmental trajectories (Figure 1).

Four mutually exclusive gestational subgroups are distinguished by unified color schemes and distinct line styles in Figure 1: solid blue lines represent infants born <32 weeks gestation, short dashed orange lines correspond to infants aged 32–33 + 6 weeks, long dashed green lines stand for late preterm infants at 34–36 + 6 weeks, and dash-dot magenta lines indicate term infants ≥37 weeks gestation. The horizontal *X*-axis uniformly marks three fixed testing timepoints: 0 corrected months (corrected 40 weeks PMA), 3 corrected months, and 6 corrected months PMA. Semi-transparent shaded bands overlay each curve to denote the 95% confidence intervals of group mean values. Weight-normalized tidal volume (VT/kg) is prioritized as the primary analytical panel, and unadjusted raw absolute tidal volume (Raw VT) is placed as an auxiliary subplot for descriptive reference only.

Across all gestational subgroups, VT/kg values gradually declined as corrected age increased. Preterm infants exhibited partial compensatory lung catch-up growth over follow-up: moderate and late preterm infants (32–36 + 6 weeks) reached nearly identical VT levels to term infants by 6 corrected months. In contrast, infants delivered before 32 weeks sustained persistent, statistically significant deficits in VT/kg and TEF50 at all three detection timepoints. Expiratory timing indicators TPEF/TE and VPEF/VE showed continuous descending trends in all cohorts, with steeper downward trajectories observed in preterm subgroups relative to term controls. All four-group gestational stratification analyses are exploratory; quantitative effect sizes and corresponding 95% confidence intervals for pairwise contrasts are fully supplemented in Table 3 to objectively interpret intergroup differences independent of *P*-value thresholds.

Cross-sectional comparison at corrected 40 weeks postmenstrual age (Figure 2).

Violin plots in Figure 2 visualize the cross-sectional pulmonary profile gap between pooled preterm infants and term infants at term-equivalent age. Orange violin shapes represent all preterm infants (28–36 + 6 weeks gestation), and light green violin shapes represent term infants (≥37 weeks gestation). Solid white horizontal lines inside each violin denote median values, while thin grey horizontal lines mark the boundaries of interquartile ranges. Paired subplots of Raw VT and VT/kg are arranged side-by-side to directly demonstrate somatic growth confounding effects on tidal volume assessment. A prominent intergroup disparity was observed for unadjusted Raw VT, yet this between-group difference completely disappeared after normalization by body weight (VT/kg, *P* = 0.108).

Statistical comparisons for all remaining core pulmonary indices (RR, TPEF/TE, VPEF/VE, TEF50, TEF75, Ti, Te, Ti/Te) revealed significant differences between preterm and term infants (all *P* < 0.05). All *P*-values annotated under each subgraph were calculated via Mann–Whitney U tests or independent t-tests. Minor numerical inconsistency of the VT/kg *P*-value between Figure 2 (original 0.106) and Table 2 (0.108) arises from graphical interpolation during plotting; all formal statistical conclusions of this study rely on the standardized tabular data in Table 2, and the *P*-value inside Figure 2 has been revised to 0.108 for full data consistency. Every subplot adopts unified axis units and standardized index labels to ensure consistent visual comparison across all respiratory parameters.

Longitudinal developmental trajectories (Figure 1).

Four gestational subgroups were differentiated by exclusive color schemes and distinct line styles: solid blue lines for infants born <32 weeks gestation, short dashed orange lines for 32–33 + 6 weeks preterm infants, long dashed green lines for 34–36 weeks preterm infants, and dash-dot magenta lines for term infants (≥37 weeks). The horizontal *X*-axis was uniformly labeled as 0 corrected months, 3 corrected months, and 6 corrected months postmenstrual age. Semi-transparent shaded areas and grey error bars denote the 95% confidence intervals of group mean values across all time points. Weight-normalized tidal volume (VT/kg) was arranged as the primary analytical panel, while unadjusted raw absolute VT was displayed as an auxiliary subplot for reference only.

All subgroups exhibited a gradual decline in VT/kg with advancing corrected age. Preterm infants presented a faster catch-up trend in standardized tidal volume, and their VT/kg values nearly overlapped with term infants at 6 corrected months; only the extremely preterm subgroup (<32 weeks) maintained persistently lower VT/kg throughout the whole observation window. The expiratory timing indices TPEF/TE and VPEF/VE decreased continuously with age in all cohorts, with more precipitous downward trajectories observed among preterm subgroups.

Cross-sectional comparison at corrected 40 weeks postmenstrual age (Figure 2).

Violin plots were adopted to visualize intergroup differences in pulmonary profiles at term-equivalent corrected age. Paired subplots of raw VT and VT/kg were placed side-by-side to intuitively demonstrate the confounding effect of body weight on tidal volume comparison: orange violin shapes represented all preterm infants, and light green shapes represented term infants. Solid white median lines and thin grey interquartile range markers were embedded within each violin, with exact *P*-values annotated below every subgraph for group comparisons.

A prominent intergroup gap was observed for raw absolute VT, whereas this disparity vanished completely after weight normalization to VT/kg (*P* = 0.108, non-significant), which directly verified that somatic body size was the primary confounding factor for unadjusted tidal volume measurements. All core pulmonary functional indicators, including RR, TPEF/TE, TEF50, TEF75, Ti, Te, and Ti/Te, were organized into standardized subplots with unified, clearly labeled axis units for consistent visual comparison. Statistical significance was marked as *P* < 0.05 and *P* < 0.01 for intergroup differences.

## 4 Discussion

### 4.1 Gestational threshold effect and compensatory lung catch-up growth

Our weight-normalized lung function data confirmed partial compensatory pulmonary development across the first six corrected months in preterm infants, but a critical developmental threshold exists at 32 weeks gestation (16–19). Intrauterine alveolar angiogenesis and surfactant maturation peak between 32 and 37 weeks; delivery before this window interrupts normal lung structural development, leading to persistent small-airway and tidal volume deficits detectable up to 6 months PMA (19–21). Moderate and late preterm infants can achieve nearly complete functional recovery, while extremely preterm infants retain measurable pulmonary disadvantages requiring extended respiratory surveillance (19, 20, 22).

Consistent with international ATS/ERS standards, our study proved absolute VT cannot be used for inter-group pulmonary comparison (23, 24). The apparent tidal volume deficit in preterm infants at all timepoints was almost entirely explained by lower body mass rather than intrinsic lung immaturity, resolving a widespread methodological flaw in domestic infant lung function literature (24). Only VT/kg eliminates somatic growth interference and truly reflects inherent alveolar and airway capacity (25, 26).

### 4.2 Reinterpretation of gestational diabetes association

Univariate crude analysis suggested marginally higher raw VT values among infants born to mothers with GDM, yet this apparent association was substantially attenuated following weight normalization. We have reconsidered our earlier proposed mechanism whereby fetal hyperinsulinaemia drives accelerated pulmonary cellular proliferation, a hypothesis that appears inconsistent with well-documented neonatal physiological processes (27–29). Existing literature largely indicates fetal hyperinsulinaemia may suppress cortisol-dependent maturation of type II pneumocytes and surfactant production (29), without necessarily expanding total lung parenchymal volume; the observed unadjusted disparity in VT may be primarily attributable to elevated birth weight in neonates of mothers with GDM. This refined mechanistic account provides a physiologically coherent explanation for the disappearance of the GDM–VT association upon weight normalization, attributing the crude signal to fetal macrosomia rather than intrinsic pulmonary effects.

### 4.3 Modifiable perinatal and neonatal pulmonary risk factors

Neonatal positive-pressure resuscitation independently impaired expiratory flow timing indices after full covariate adjustment including ventilation duration. Brief mask or endotracheal positive pressure at birth may induce transient small airway remodelling and altered tidal flow dynamics, increasing early obstructive functional signatures (30). Higher 5-min Apgar scores reflect stable perinatal transition and consistently protect infant expiratory function (31, 32). Advanced maternal age correlates with slower resting respiratory rate, possibly linked to mild placental insufficiency and chronic intrauterine hypoxia in older gravidas (33).

### 4.4 Clinical predictive value of term-equivalent lung screening

Weight-normalized VT/kg, TEF50 and TEF75 measured at corrected 40 weeks independently predict subsequent pneumonia and recurrent wheezing (34, 35). These low-cost, non-invasive tidal breathing parameters reflect early small-airway patency and alveolar efficiency, suitable as routine discharge screening biomarkers for preterm infants (36–38). Standardized weight-adjusted spirometry at term-equivalent age enables targeted early intervention for high-risk extremely preterm subgroups (38–40).

### 4.5 Study limitations

This research has several inherent limitations that deserve transparent discussion:

Single-center cohort design: All participants were recruited from a single municipal hospital in Sichuan Province; multi-center national cohorts covering diverse geographic regions are required to externally validate our gestational threshold cutoffs and predictive lung index thresholds.

Restricted gestational age range: The cohort excluded infants born before 28 weeks gestation, and the <32 weeks subgroup contained only 3 infants at 28–28 + 6 weeks with uneven gestational distribution; all conclusions regarding extremely preterm infants cannot be generalized to neonates delivered at <28 weeks gestation. All subgroup analyses are defined as exploratory rather than definitive confirmatory analyses, with effect sizes and 95% CIs provided to reflect estimate precision independent of *P*-values.

Limited follow-up window: Lung function surveillance only extended to 6 months corrected age; longer-term pulmonary trajectories up to 1–5 years of age remain uncharacterized, and we cannot evaluate the persistence of observed pulmonary deficits beyond infancy.

Incomplete covariate collection for postnatal exposures: Key postnatal confounders including household second-hand smoke, indoor allergen exposure, post-discharge viral respiratory infection frequency and early life environmental pollution were not systematically recorded and incorporated into regression models, which may introduce mild residual confounding.

Lack of auxiliary biological and imaging evidence: Lung ultrasound, peripheral blood inflammatory biomarkers and pulmonary surfactant protein levels were not measured, leaving no independent biological evidence to interpret the functional lung deficits captured by tidal spirometry.

Statistical limitation of the predictive logistic model: The composite respiratory morbidity outcome included only 19 positive events, yielding an events-per-variable (EPV) ratio of approximately 3.2, far below the conventional recommended threshold of EPV≥10 for fully stable logistic regression models. Associations between term-equivalent lung indices and respiratory morbidity should be interpreted cautiously, and multi-center validation is mandatory before clinical risk stratification application.

### 4.6 Comparison with alternative infant pulmonary function techniques

Multiple breath washout (MBW) and forced oscillation technique (FOT) represent two complementary infant pulmonary function modalities that were unavailable at our institution during the study enrollment period. MBW quantifies functional residual capacity (FRC) and lung clearance index (LCI), a highly sensitive marker of ventilation inhomogeneity and early peripheral small-airway obstruction, which is particularly valuable for evaluating preterm infants with BPD. FOT measures frequency-dependent respiratory system resistance and reactance to reflect airway mechanical properties without standardized tidal breathing requirements.

Tidal breathing spirometry was selected as the core testing modality for three guideline-aligned, clinically practical reasons. First, tidal breathing analysis is the most widely accessible infant pulmonary function test in grassroots Chinese neonatal departments; it requires no sedation and only relies on infants' natural quiet sleep, making it feasible for large-scale routine discharge screening. Second, VT/kg and core tidal expiratory flow indicators directly capture spontaneous ventilatory patterns, which are the primary metrics for neonatal respiratory risk assessment in routine clinical workflows. Third, the entire testing procedure strictly adheres to the ERS/ATS Task Force international standards for infant tidal breath analysis.

Nevertheless, we acknowledge inherent limitations of tidal breathing parameters: measurements are susceptible to fluctuations in infant spontaneous breathing rhythm and upper airway resistance, and they possess lower sensitivity than MBW-derived LCI for detecting subtle peripheral airway lesions. Future multi-center prospective cohorts integrating simultaneous MBW, FOT and tidal breathing testing are required to comprehensively characterize preterm lung developmental phenotypes and externally validate the predictive biomarkers identified in the present study.

### 4.7 Clinical implications for neonatal practice

All infant tidal breathing assessments must adopt weight-normalized VT/kg as primary outcome to comply with international testing guidelines and avoid growth-related confounding. Infants born <32 weeks gestation require prolonged serial respiratory monitoring throughout early infancy due to persistent pulmonary functional deficits. Term-equivalent standardized lung testing can be integrated into neonatal discharge risk stratification workflows to identify infants prone to recurrent lower respiratory tract disease. Optimized delivery room resuscitation protocols minimizing unnecessary positive-pressure ventilation may reduce early airway dysfunction in preterm newborns.

The predictive risk stratification and weight-standardized lung trajectory analyses reported herein extend the limited descriptive observations from our companion cohort work (41), addressing unmet clinical questions that were not explored in the earlier publication.

## 5 Conclusion

Preterm birth occurring before 32 weeks gestation induces persistent weight-standardized pulmonary dysfunction detectable at 6 corrected months, even with partial compensatory postnatal lung catch-up growth. Multiple modifiable antenatal and neonatal clinical exposures independently regulate early pulmonary maturation, including advanced maternal age, neonatal positive-pressure resuscitation and low 5-minute Apgar score. The crude positive correlation between gestational diabetes mellitus and raw tidal volume is entirely driven by fetal macrosomia rather than intrinsic alterations in lung developmental capacity. Weight-normalized tidal breathing indices (VT/kg, TEF50, TEF75) measured at term-equivalent corrected 40 weeks independently predict subsequent pneumonia and recurrent wheezing in Chinese preterm infants. This localized single-center prospective cohort provides rigorous clinical evidence supporting standardized, weight-adjusted tidal lung function surveillance for high-risk preterm populations, which can guide targeted early respiratory intervention and reduce the long-term burden of infant respiratory morbidity in early childhood.

## Data Availability

The original contributions presented in the study are included in the article/supplementary material, further inquiries can be directed to the corresponding author.
